# Supplementary figures and images for: Characterization of depth perception information inferred from neuronal activity in primary visual cortex
Source: PLoS One. 2025 Aug 7;20(8):e0329788. doi: 10.1371/journal.pone.0329788 (PMC12331029; doi:10.1371/journal.pone.0329788)

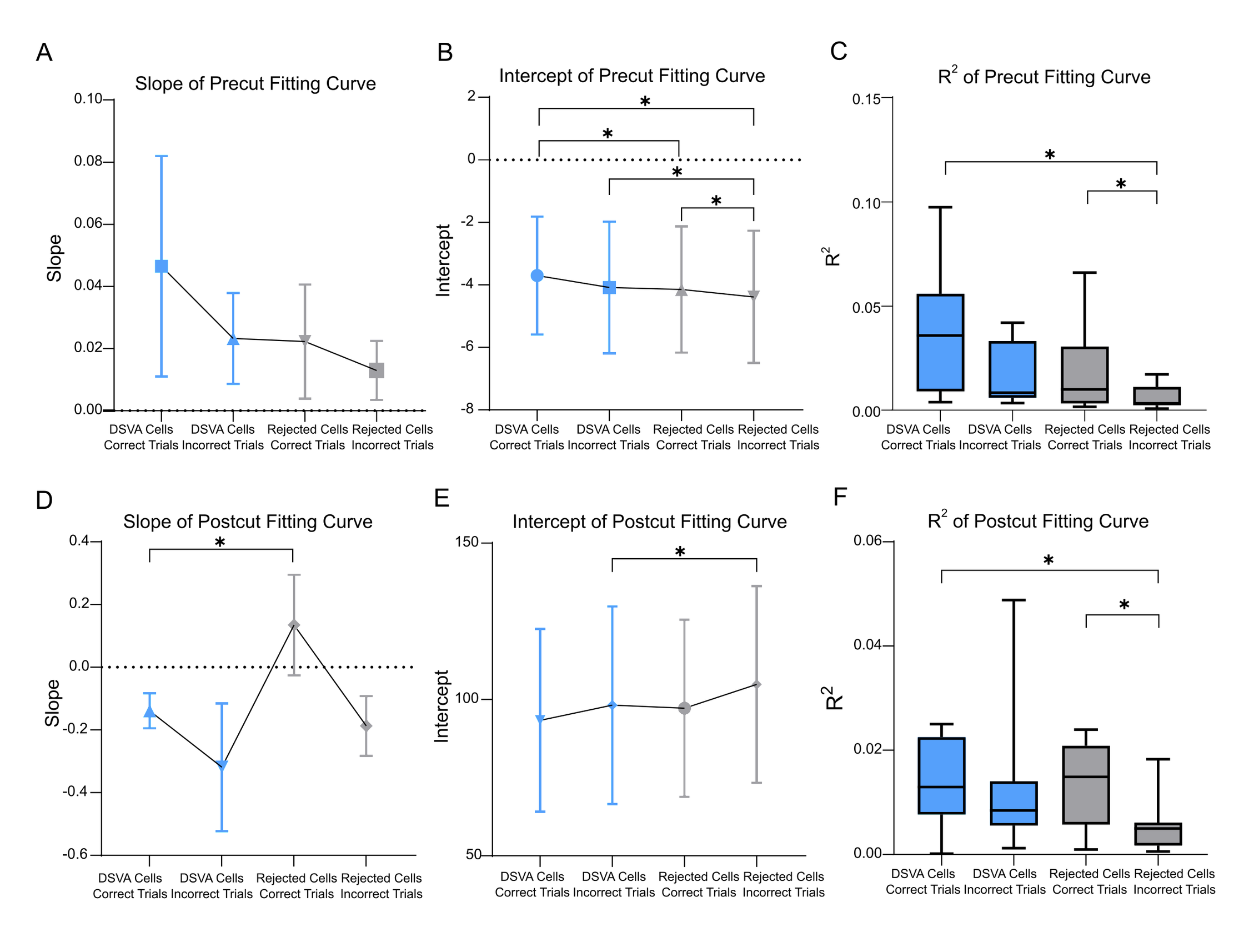

Supplement: S2 Fig — Four combined categories show significant differences by statistic markers. Different markers represent different categories. (A) Slopes of the fitted lines before time point 0 for all subjects. Different markers represent different categories. Error bars represent SEM for all subjects. (B) Intercepts of the fitted lines before time point 0 for all subjects. Error bars represent SEM for all subjects. Mann-Whitney test, *p < 0.05. (C) R2 of the fitted lines before time point 0 for all subjects. Error bars represent SEM for all subjects. The top and bottom edges of the box represent the maximum and minimum value, respectively. Mann-Whitney test, *p < 0.05. (D) Slopes of the fitted lines after time point 0 for all subjects. Different markers represent different categories. Error bars represent SEM for all subjects. Mann-Whitney test, *p < 0.05. (E) Intercepts of the fitted lines after time point 0 for all subjects. Error bars represent SEM for all subjects. Mann-Whitney test, *p < 0.05. (F) R2 of the fitted lines after time point 0 for all subjects. Error bars represent SEM for all subjects. The top and bottom edges of the box represent the maximum and minimum value, respectively. Mann-Whitney test, *p < 0.05. (TIF) [file pone.0329788.s002.tif]

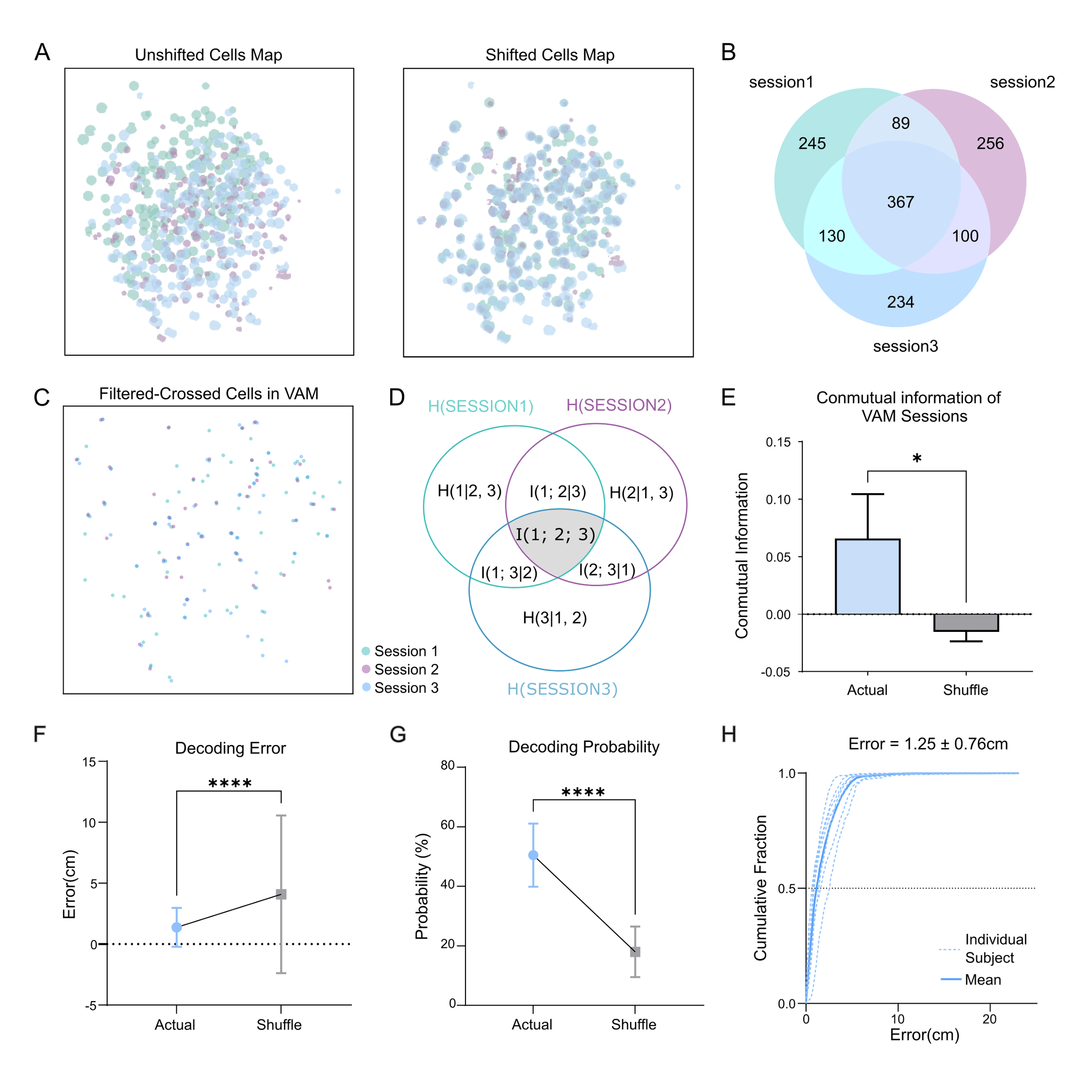

Supplement: S3 Fig — (A) Cell maps showing unshifted and shifted cell positions for a sample subject. The unshifted map shows original cell positions across three VSLM sessions (left), while the shifted map shows positions adjusted by parameters calculated through cross-registration. Different colors indicate sessions. (B) Venn diagram illustrating the overlap of crossed cells across three VSLM sessions with 367 cells identified as crossed. (C) Positions of DSVA cells within crossed cells from three VSLM sessions for a sample subject.; each dot represents a cell. (D) Schematic of conditional mutual information for cells filtered across three VSLM sessions. H (X ∣ Y, Z) is conditional entropy of variable X given Y and Z; I (X; Y ∣ Z) is conditional mutual information between X and Y given Z. I (X; Y; Z) is the joint mutual information of variables, X, Y, and Z. (E) Conditional mutual information of DSVA cells across three VSLM sessions compared to shuffled data. Error bars represent STD of individual data. The conditional mutual information of the actual data is significantly higher than shuffled data. Wilcoxon test, *p < 0.05. (F) Decoding errors of predicted positions for actual data vs. shuffled data; actual data are significantly smaller than shuffled data. Mann-Whitney test, ****p < 0.0001. (G) Decoding probabilities for predicted positions in actual data vs. shuffled data; actual data probabilities are significantly higher. Mann-Whitney test, ****p < 0.0001. (H) Cumulative fraction of decoding errors across subjects, with a median decoding error of 1.25 ± 0.76 cm. Dashed line show individual subjects; solid line represents mean cumulative fraction of decoding errors. (TIF) [file pone.0329788.s003.tif]
