# Supplementary material for: Characterization of depth perception information inferred from neuronal activity in primary visual cortex
Source: PLoS One. 2025 Aug 7;20(8):e0329788. doi: 10.1371/journal.pone.0329788 (PMC12331029; doi:10.1371/journal.pone.0329788)
Supplement: Attachment — Submitted filename: Response to Reviewers.docx [file pone.0329788.s004.docx]

**Response to Reviewers' Comments**

**PONE-D-24-51051**

**Characterization of Depth Perception Information Inferred from Neuronal Activity in Primary Visual Cortex**

Dear Reviewers,

Thank you for your valuable advice. Your suggestions have been extremely beneficial to us. We sincerely appreciate the time and effort you have dedicated to reviewing our manuscript. Below, we provide a point-by-point response to each concern raised.

Reviewer #1

*This paper reports investigations on how primate-V1 could play complex, higher-level role beyond our imagination upon its basic visual processing, i.e., passive and active depth-perception to constitute a functional segregation. The paper is well written and gradually illustrating the main results through experiments on mouse. The results are of importance to further understand the unexplored roles of V1 visual cortex in visual processing tasks. From my perspective, it is a high-quality paper deserves to be published in the journal of PLOS ONE.*

We are deeply grateful for the reviewer’s positive evaluation and endorsement of our study. The acknowledgment of the paper’s quality and relevance to understanding V1’s higher-level roles is highly encouraging.

Reviewer #2

*In the current manuscript, Dong et al. investigated an important question: what are the specific contributions of neurons in primary visual cortex in various aspects of depth perception. In this manuscript, the authors trained mice to perform two tasks: visual cliff (passive depth perception) and variable-length-arm maze (active depth discrimination). They recorded in vivo calcium imaging in freely moving mice, and the constrained non-negative matrix factorization algorithm to identified depth-related V1 neurons in two tasks (DSVA and DSVC cells). They showed that specific groups of V1 neurons are selectively activated, suggesting functional segregation. Moreover, V1 neurons prefer encoding objective positions rather than egocentric distances in non-depth-based tasks.*

*The manuscript is written clear. The analyses are sound, and the results are generally interesting. I have however a few concerns, and hope the author will be able to clarify those issues.*

We thank the reviewer for their thorough and insightful comments. Below are our detailed responses:

Comment 1: *The definition of DSVA cell was introduced at Page 11 Line 210, but DSVA cell firstly appeared at Page 18 Line 334. This may confuse the readers, please modify.*

Response: We agree that the initial presentation of DSVA cells could cause confusion. In the revised manuscript, we introduced the term "DSVA cells" with its definition when first mentioned (Page 12, Line 217).

Comment 2: *In Variable-Arms Maze (VAM) task, the number of arms can be changed. In this manuscript, the authors designed a radial variable-length-arm maze, required mice to utilize depth information actively in decision-making. This task was more specific, and should be distinguished from other VAM tasks. I suggest using VSLM, instead of VSM, to define this task.*

Response: We appreciate this suggestion. To avoid ambiguity with general VAM tasks, we replaced all statements of "VAM" with "VSLM" (Variable Spatial Length Maze) throughout the text.

And we added an explanation in the Methods section (Page 7, Line 125) distinguishing VSLM from other VAM paradigms.

Reviewer #3

*Since I have suggested to accept the manuscript, I have no more review comments to the author.*

*I have no more explanation for my answers to the questions above or additional comments for the authors.*

We sincerely thank the reviewer for the supportive assessment and recommendation for acceptance.

We hope these revisions and responses have adequately addressed all concerns. Thank you again for the opportunity to improve our work. We look forward to your further guidance.

Sincerely,

Dr. Haibing Xu

Professor

Guangdong-Hong Kong-Macao Greater Bay Area Center for Brain Science and Brain-Inspired Intelligence

Southern Medical University, Guangzhou, China

Email: haibingxu@smu.edu.cn
